# The Hospital. Nursing Section

**Published:** 1906-03-31

**Authors:** 


### The Hospital.

**Nursing Section.**

Contributions for "THE HOSPITAL," should be addressed to the EDITOR, "THE HOSPITAL" NURSING SECTION, 28 & 29 Southampton Street, Strand, London, W.C.

No. 1,018.—VOL. XXXIX. SATURDAY, MARCH 31, 1906.

### Notes on News from the Nursing World.

### THE NEW ARMY MATRON-IN-CHIEF.

MISS C. H. KEER, R.R.C., has been appointed Matron-in-Chief of Queen Alexandra's Imperial Military Nursing Service and will enter upon her duties on Wednesday next, when Miss Sidney Browne retires. Miss Keer has been in the Service for eighteen years. She was trained in the United States at Boston City Hospital, and left in 1887 to enter the Army the same year. She was stationed for one year at Netley, for five years and three months in Egypt, and for five and a half years at Dover. She was then, in 1899, sent to South Africa with No. 4 General Hospital. From that hospital she was transferred to No. 4 Stationary Field Hospital then at Spearman's Camp, which followed General Buller's forces through Natal to Standerton, Transvaal. She was then sent from Standerton to take charge at Howick, Natal, and finally returned to England in 1902 after the war was over. Miss Keer was next stationed at Colchester Military Hospital in charge of the nursing staff, and while she was there she entered Queen Alexandra's Imperial Military Nursing Service, being appointed Principal Matron in South Africa. In June 1903 she sailed for South Africa, returning to this country last month as Matron-in-Chief designate, and she has lately been engaged at the War Office familiarising herself with her new duties. The decoration of the Royal Red Cross was bestowed upon her by the King in 1902 after her return from South Africa. Miss Keer is to be succeeded as Principal Matron in South Africa by Miss F. E. Addams-Williams, R.R.C., matron of the Royal Victoria Hospital at Netley, who has been placed under orders to proceed to Pretoria. Miss Addams-Williams has been in the Service two years longer than Miss Keer. She was trained at the Royal Infirmary, Edinburgh, and since she entered the Army Service has been superintendent in charge at the Military Hospital, Canterbury, and the Connaught Hospital, North Camp, Aldershot. The important post of matron at Netley has been assigned to Miss A. B. Smith, R.R.C., matron, Royal Infirmary, Dublin.

### THE IMPERIAL MILITARY NURSING SERVICE.

WE are officially informed that Miss G. A. Aitchison and Miss A. M. Phillips have been appointed staff nurses in Queen Alexandra's Imperial Military Nursing Service. Miss S. L. Wilshaw, R.R.C., and Miss M. M. Blakely, sisters, have been transferred to the Military Hospital, Cairo, Egypt, from the Military Hospital, Khartoum; and Miss W. G. Massey, sister, to the Military Hospital, Khartoum, from the Military Hospital, Cairo. Miss E. G. Barrett, staff nurse, has been transferred to the Military Hospital, Portsmouth, from the Royal Infirmary, Dublin; and Miss G. M. Smith, staff nurse, to the Military Hospital, Khartoum, from the Military Hospital, Cairo, Egypt. Miss M. M. A. McCreery, staff nurse, has been confirmed in her appointment.

### PRINCESS CHRISTIAN AND HER NURSE.

AT the opening of the Queen Victoria Memorial Hospital, Mont Boron, Nice, by Princess Christian the other day, Her Royal Highness was received and conducted round the buildings by the matron, Miss Shappera, accompanied by Miss Warcup, matron of the Riviera Nurses Co-operation, Nice, and six of her staff who were assisting. Miss Warcup was honoured by a shake of the hand and "Here is one of my nurses" from the Princess, who observed that she was wearing the medal of the Army Nursing Service Reserve. A feature of the hospital is a garden where convalescents will be able to sit and enjoy the sunshine. There are thirty beds, of which eight are free, the others being for paying patients.

### PRINCESS HENRY OF BATTENBERG AND DISTRICT NURSING.

THE annual meeting of the Ryde District Nursing Society was held on Monday, Princess Henry of Battenberg presiding. The Princess, who is President of the Society, was received by the Mayor on her arrival at the Town Hall. In the report, which was read by the Secretary, it was stated that the nurses had paid over 8,000 visits during the year and had attended nearly 500 new cases. The thanks of the meeting were given to the Princess for her interest in the organisation, and congratulatory reference was made to the engagement of Princess Ena.

### FEVER TRAINING AND GENERAL HOSPITALS.

IN an interview by our Commissioner with the matron of the Park Hospital, Hither Green, which is reported elsewhere, a notable suggestion was made by Miss Thomas. She thinks that it would be an exceedingly good thing if the general hospitals could be affiliated to fever hospitals of recognised standing so that during their training the probationers of the former could be sent to the latter for a period of six months. In her opinion, the experience thus gained would give the probationers in training at the general hospital a good idea of fever nursing, and would show them how fever nursing has progressed during the last twenty-five years. The suggestion is worthy of consideration, and we think that a perusal of the conditions of nursing at the Park Hospital, which it is fair to say are mainly due to the action of the Metropolitan Asylums Board, influenced, no doubt, by the trend of opinion and the example of the general hospitals, will impress many favourably as to the advantages to be gained. Whether the idea is practicable is another matter, and on this point we shall be glad to learn the views of matrons and sisters of general hospitals.

### MONEY PRESENTATIONS.

From time to time we record in our columns presentations to matrons and nurses on the occasion of their retirement from the sphere of duty in which they gained the goodwill of those associated with them; and we are disposed to agree with a matron who writes to us with the view of checking what she calls the liability of people to get into "ruts" in the selection of their gifts. Dressing and travelling bags, toilet sets, and writing cases, are all very well in their way, but when we hear of a nurse who has received three of the last it seems time to make a new departure. We therefore think that the suggestion that works of art would be an acceptable variation will be welcomed and noted, especially as it is exceedingly easy to regulate the price for pictures according to the sum subscribed. But with regard to the presentation of money, which our correspondent favours, we are utterly opposed to it as a matter of principle, at any rate so far as hospital and private nurses are concerned. Both of these should be adequately remunerated for their work, and there is no reason why their salaries should be supplemented by a sort of douceur. A kindly recognition on the part of the authorities and of colleagues, by the presentation of a suitable memento, is quite a different thing.

### PARLIAMENT AND ASYLUM WORKERS.

We understand that upwards of sixty members of the House of Commons have undertaken to support the legal recognition of the claims of public asylum workers to pensions. But the question cannot be said to be ripe for legislation until the Executive of the Asylum Workers' Association have authoritatively ascertained the views of the members on several important issues. It should be known, for example, how far they approve of a scheme in which compulsory contributions—by way of a percentage deduction from wages—would form a part; whether, in this case especially, security of tenure of office after a certain term of approved service should be guaranteed; and whether the aggregate of service, although under different asylum authorities, should be taken into account in computing pensions. These are issues which the Committee have discussed, but as they themselves desire to learn more fully the opinions of the members of the Association, we do not see how legislation could be usefully proposed at present.

### THE WEARING OF UNIFORMS OUT OF DOORS.

According to the *American Journal of Nursing*, "it is only nurses of the second and third rate, or no rate at all, who are seen about in street-cars, stores, restaurants, and theatres in their nursing costume." That may be so in the United States, but in England, at least three classes of nurses, neither necessarily second nor third rate, wear uniform out of doors. There are the nurses who, from motives of economy of time or money, do not change into private dress when they are off duty, and those whose whole life is so merged in their avocation that they never think of wearing any costume than their uniform. Then, of course, there are the Army sisters who are required by the regulations to invariably appear in uniform. Certainly, the slur which is cast upon the wearers of nursing costume out of doors in the United States by the American magazine could not be thrown at those who follow the practice on this side of the Atlantic.

### CANADIAN FIREMEN'S GRATITUDE TO AN ENGLISH NURSE.

A very interesting incident took place at Fort Frances Rectory, Ontario, the other day, when one of the brotherhood of firemen and locomotive engineers waited upon Miss Annie Barratt, a district nurse who was staying at the Rectory, and presented her with an address and a sapphire ring. The presentation, which was made on behalf of the brotherhood, was in recognition of the services rendered by Miss Barratt, who in August last nursed several of the firemen who were severely scalded near Bears Pass. Miss Barratt, who has been district nurse at Fort Frances for the past year, was formerly night superintendent at Plaistow Hospital, and sister at Birmingham Poor-law Infirmary.

### A PRIZE FOR VICTORIAN ARMY SISTERS.

A competition examination for a prize of £10 10s. was held at the Victoria Barracks, Melbourne, on the evening of Friday, February 15. Sixteen out of twenty-four sisters entered for the examination, which consisted of three written questions and two bandaging demonstrations. The sisters all appeared in their military uniform for the first time, and looked very trim and smart. It is very similar to that worn in Queen Alexandra's Service—dress, cape, and bonnet of grey; scarlet cape and white cap for indoor wear—the difference being in the facings of chocolate, which is the colour adopted by the medical service in Australia.

### A BADGE FOR IRISH NURSES.

At the third anniversary meeting, last week, of the Irish Nurses' Association, it was decided that the members should adopt a distinctive badge, and a prize was offered by two members for the best design. Miss Hampson, having given a brief retiring address in which she outlined the work done during the year, Miss Kelly took the chair as president. Mrs. Kildare-Treacey was elected Vice-President by a large majority of votes.

### UNION HOSPITAL, NEWCASTLE-UPON-TYNE.

On Wednesday last week, an interesting ceremony took place in the Union Hospital, Newcastle-on-Tyne. At a recent examination for the nursing certificate, four of the probationers who had successfully passed a final examination after the usual three years' course, were presented with certificates, namely, Nurses Colling, Wilson, Poad, and Harrison. The late Dr. Heath, of Newcastle-upon-Tyne, by his will left a legacy, the proceeds of which were to be devoted to nurses trained in the hospitals of Newcastle-upon-Tyne, as prizes, and three of these nurses accordingly received them. The examinations were conducted by Dr. W. D. Arnison, of the Royal Infirmary, who expressed his great satisfaction at the knowledge shown by the nurses. The prizes were presented by Dr. Ranken Lyle, master of the Maternity Hospital and Lecturer at the College of Medicine. This function concluded, a number of guests were entertained to tea.

### "POUND DAY" AT THE BELGRAVE HOSPITAL FOR CHILDREN.

The second "Pound Day," in connection with the Belgrave Hospital for Children, at Kennington, was held on Saturday. The experiment made by the matron last year was so successful that at the end of the year she found the stores supplied on the occasion still unexhausted. On this occasion the pounds of foodstuffs, chiefly of the dried goods order, amounted in weight to a ton and a half. There were many pounds of cake, sweetmeats, and confections, but tea was quite the predominating article selected.

### DEPENDENCE UPON "ACCIDENTAL" INCOME

The otherwise satisfactory report, which was adopted at the annual meeting of the Taunton District Nursing Association, is marred by the fact that the amount expended exceeded by £8 the sum received. Moreover, while it is a matter for congratulation that the Association derived substantial help from Easter sports, a Volunteer Band concert, a Nelson centenary, and a carnival, we think that one of the speakers wisely remarked that too much dependence was placed upon what he might call "accidental" income. Of course, this tendency is not confined to the Taunton Association, and nursing organisations are not only justified in availing themselves of unusual aids to revenue, but are bound to try and secure them. Yet the aim always in view should be to cover the regular working expenses by annual subscriptions, "accidental" income being used for establishment outlay, or invested as capital against a day of need.

### POOR-LAW NURSING IN THE NORTH.

A comparison made by Mr. Jenner Fust, Inspector for Lancashire and parts of Cumberland and Westmorland, in his last report on nursing in workhouses is interesting. The number of sick on January 1, 1905, was 10,840, who were looked after by 906 nurses and 164 pauper attendants; whereas ten years previously the number of sick was 8,830, with 505 nurses and 299 pauper attendants. Mr. Philip Bagenal, in his annual report to January 1, 1905, as Inspector for the Union Counties of the East and West Riding of Yorkshire, states that the number of nurses on day duty in 1903 was 314, and on night duty 109, making a total of 423. In 1904 the number was 433 nurses. This works out at a rate of 11.95 patients per nurse, as against 11.69 in 1903, only a slight improvement, although an indication of progress. There were still no fewer than 42 pauper attendants employed in the sick wards.

### WORKPEOPLE'S COLLECTIONS.

A passage in the report which was read at the annual meeting in connection with the Wednesbury District Nursing Institute merits attention. During last year the main feature was, as the Chairman pointed out, the increase of £15 in the amount collected from the workpeople. In the report it is stated that this feature is most gratifying, "because there is no doubt that the future of the institute will in a great measure depend upon the income from this source." We can readily believe that this view of the situation is accurate. Moreover, the position in Wednesbury is much the same as in many other manufacturing centres, and we are satisfied that in days to come it will be essential to tap in a far more systematic manner than is often done now, a source of income which ought, within limits, to prove inexhaustible. We are persuaded that, as a rule, the only reason why workpeople who are regularly employed do not support the nursing organisations to which they owe so much, is want of organisation, combined with lack of energy. Wherever these two defects are remedied, we venture to predict satisfactory results.

### DISTRICT NURSING AMONGST THE JEWISH COMMUNITY.

It is interesting to observe that the Jewish community adhere to the title of Sick Room Helps Society for a movement which was founded many years ago having for its object maternity nursing and the care of the home among the Jewish poor. Under the auspices of this excellent organisation, a Jewish Nurses' Home was consecrated by the Chief Rabbi on Thursday last week. Here the Jewish nurses will live and receive training, and on behalf of this development appeals for financial help are made which we do not doubt will meet with a characteristically ready response. District nursing amongst the Jews is not any the less appreciated because it is carried on under the direction of the Sick Room Helps Society.

### SHORT ITEMS.

Special seats were reserved in Melbourne Cathedral at the Memorial Service held on Sunday, February 18, in memory of the late King of Denmark, for the lady superintendent and matron of the Victorian Army Service.—The result of the lecture given by Miss Milner at Grosvenor House in aid of the Ober-Ammergau Nursing Fund has been to bring the amount within £10 of the required sum.—On behalf of the Maternity Charity and District Nurses' Home at Plaistow, a concert will be given at Stafford House on the afternoon of Thursday, May 10.—The superintendent of the District Nurses' Home, Leeds, sends us the annual report of the Leeds Association for Nursing the Sick Poor in their Homes, from which we learn that the Secretary stated that the amount spent on tram and railway fares was "about half the cost of a nurse," not "' sufficient to support a nurse for a year."

### The Nursing Outlook.

"From magnanimity, all fears above;
From nobler recompense, above applause,
Which owes to man's short outlook all its charm."

### WANTED A POLICY

We have no doubt that the Lord President is fully aware that the deputation which he received a few weeks ago was not representative, in any real sense, of the interests which will have to be mainly considered in any Act for the registration of trained nurses. There is no precedent for taking the control and management of the education and training of any class of students out of the hands of the principals and teachers who have had it in charge previous to legislative action. Imagine the state of chaos and breakdown which must result from an Act of Parliament which would ignore the universities and medical schools when dealing with the registration of medical practitioners! Yet in principle, and indeed in fact, the Bills hitherto submitted to Parliament have ignored the present teachers and instructors of nurses, and have failed to provide directly or in any real sense for the representation of the nurse-training schools. The Lord President is no doubt anxious to be approached by the authorities of the nurse-training schools and the teachers and instructors of probationers throughout the country, in order that he may have before him what he has failed to elicit at present, the matured opinion, based upon experience, of those who have the right to speak with the greatest authority in regard to the legislation, discipline, education and training of probationers, and the regulation of the nursing service throughout the country. The sooner, therefore, steps are taken by those immediately responsible to interview the Lord President and supply him with adequate official and reliable information, the better.

The crux of the present position is the absence of a Bill which has the collective support of the nurse-training schools as well as of the teachers and trainers of probationers. Hitherto the majority of these authorities have contented themselves by opposing the registration of nurses in any form or shape. This attitude has been taken advantage of by their opponents, who have displayed more practical wisdom by endeavouring to attract outsiders and to so form public opinion in favour of the Registration Bills. There can be no doubt that any success they may have secured is attributable mainly to the fact, that the nurse-training schools have had no policy, and so the least considered statements may have been accepted as gospel by the unthinking and ignorant. It is time that the teachers and the schools formed a General Nursing Council, with the object of circulating accurate data and information, and of guiding public opinion to secure the wisest solution of the problem presented. The time has gone by to rest the case of the schools upon a more negative backed by unyielding opposition to one particular proposal. Registration has little importance compared to a scheme which would provide for the utilisation of all the material which the various institutions, large and small, throughout the country, afford for the adequate training and thorough education of every woman who desires to become an efficient nurse. Probably Parliament may never consent to the registration of nurses, unless the nurse-training schools are entrusted with the practical control of the register and the education and instruction of probationers. Whatever party may be in power, the conservatism of our nation and Parliament usually prevents any radical disregard of precedent. Still, the nurse-training schools and their supporters should organise themselves and adopt a definite policy which rests upon ascertained facts and a knowledge of the requirements and claims of nurses.

We are glad to note that the wiser and more far-seeing leaders of the schools are beginning to realise that the time for inaction has passed; that they owe it to their responsibilities as trustees for the whole nursing body, and to the public generally, to take united action in defence of the sound principles which they represent, and that by no other means is it reasonable to suppose that the nursing outlook can be cleared and the profession of nursing in these islands freed from influences which must otherwise tend to do the cause permanent injury.

What the schools and teachers need is a leader whom they can trust, and whose record is marked by years of intelligent statesmanship which have resulted in good for the schools and all connected with them. Other countries have had little difficulty in finding such leaders when occasion has arisen, and we cannot believe that the United Kingdom, so far as nursing affairs are concerned, will be allowed to remain much longer without wise initiative, and a policy based upon wisdom and experience. It is something at any rate to know that the views we have ventured to express are gaining ground, and that there is every prospect that they are likely to receive early and careful consideration by those immediately concerned. If the nurse-training schools once agree to a progressive policy, English nursing, which owes its origin to the example and teaching of Florence Nightingale, may within her lifetime be put upon such a footing, as to make it an example of excellence, which the whole world may then be proud to imitate.

### Abdominal Surgery.

By Harold Burrows, M.B., F.R.C.S., Assistant Surgeon to the Seamen's Hospital, Greenwich, and to the Bolingbroke Hospital, Wandsworth Common.

### AFFECTIONS OF THE STOMACH.

This will be a convenient place to describe briefly one or two special methods which are employed in the diagnosis and treatment of gastric disease.

### Inflation of the Stomach.

Not infrequently it is desirable to distend a patient's stomach with air or gas in order to ascertain its position and dimensions, or to discover whether a lump in the abdomen is or is not connected with the stomach. Occasionally this inflation is effected by passing an india-rubber tube down the œsophagus into the stomach, and pumping in air until the requisite amount of distension is produced. More often, because it requires no special apparatus and is easier and less alarming to the patient, the tartaric acid and soda method is employed. The patient is first given a drachm of tartaric acid dissolved in two ounces of water, and this is followed by a drachm of sodium bicarbonate dissolved in two ounces of water. When the soda comes into contact with the acid there is an evolution of carbon dioxide gas, which causes the stomach to become distended. As soon as this has been accomplished the surgeon may wish to mark out the boundaries of the organ, so it is well to have a skin pencil at hand.

### Gastric Lavage.

Washing out the stomach is employed both as a method of treating that organ in certain cases, and to obtain its contents for purposes of analysis.

The passage of a large india-rubber tube into the stomach for the first time is an alarming and uncomfortable procedure for the patient. But if the operation has to be repeated its inconveniences tend to disappear, and after a little practice patients get very expert in passing the tube for themselves. It is not easy to introduce the tube if the patient rebels; so it is necessary to convince him beforehand that nothing very dreadful is going to happen. A few judicious words from the nurse may go a long way towards securing his confidence.

It is not necessary to describe the technique of gastric lavage, because it is a matter that concerns the surgeon rather than the nurse; but the arrangements for carrying out the operation are worth a little attention. The best position for the patient is sitting in a chair or propped up in bed. To protect his clothes a mackintosh covered by a clean towel should be fastened round his neck. The necessary apparatus should be placed on a table to the right of the patient. It will include a stomach tube, connected by a glass junction cannula with two feet of india-rubber tubing which is attached to a large glass funnel, a large jug of warm water, and some lubricant for the tube—preferably glycerine. Occasionally oil is used, but this is very objectionable to some patients, and may cause nausea and retching.

The stomach tube and funnel must be boiled immediately before use and placed in a bowl of warm boric lotion or sterilised water. A clean vessel will be required to receive the stomach contents.

It is good practice to use a graduated jug for the water which is used for washing out, so that the amount which has been introduced can be calculated at any moment and compared with the quantity which has been syphoned off.

### Test Meals.

In order to make a proper examination of the stomach it may be desirable to give the patient a test meal. There are several different kinds of test meals, and the nurse should be careful to ask for detailed instructions from the surgeon. A common and useful variety consists of a couple of slices of toast and a large cup of weak tea without sugar or milk.

Breakfast is the usual meal to select for the experiment. In certain cases it is necessary to wash out the stomach before the test meal is given.

About an hour and a half after the food has been taken the stomach contents are extracted by means of the stomach tube for examination.

The chief points in gastric technique, so far as they interest the nurse, having thus been considered, the more common operation procedures which are carried out on the stomach will be briefly discussed.

### Gastrostomy.

The object of this operation is to make a permanent opening into the stomach through which food can be introduced when the patient is unable to swallow on account of stricture of the œsophagus.

A stricture of the œsophagus does not form in a day; it is a gradual process. At first liquids and finely divided food can pass through into the stomach, only the larger portions being checked by the stricture. So that although he will probably lose weight, the patient is yet not totally incapacitated. But as time goes by the stricture gets tighter and tighter, until at last even liquid food can only be swallowed with difficulty, and the patient consequently is unable to absorb sufficient nourishment, and rapidly wastes.

For this reason he may be in a very exhausted condition by the time a gastrostomy is undertaken, and it will require all the skill attainable to tide him over the immediate effects of the operation. In many of these cases subcutaneous feeding will be required, in addition to nutrient enemata, both before and after the operation.

Apart from the complications and dangers attendant upon the operation itself, there are certain remote consequences of gastrostomy which may add very greatly to the miseries that must of necessity be caused by a permanent opening in the stomach. One of these is the escape of stomach contents through the gastrostomy opening, with the result that the skin around becomes acutely inflamed. The misery caused by this may be almost beyond endurance. The surgeon, when performing the operation, endeavours to make the opening in the stomach a valvular one, so that food cannot regurgitate through the opening. As may be easily imagined, however, the attempt is not always successful.

To lessen the ill effects when regurgitation does occur, the skin around the opening should be washed with soap and water, and then covered with vaseline, which will prevent the gastric juice coming into contact with the skin. A further precaution is to see that the patient lies down on his back for an hour or more, if possible, directly after a meal has been given, for in this position the escape of gastric juice and food is not so easy as when the patient is sitting or standing.

Some patients complain of not being able to swallow their saliva and of not tasting and biting their food. These complaints can be met by allowing them to chew their food and then to deposit it in a funnel which is connected with the gastrostomy tube, so that the food passes into the stomach.

A soft rubber catheter is perhaps the most suitable form of tube for introducing the food through the gastrostomy opening. It is only introduced at each meal-time, and is, of course, carefully cleaned after use. A short tube should never be used, as there is a danger of it slipping into the stomach.

At the time of the operation a tube may be left in the gastrostomy opening. Care must be taken that it does not slip out, as there may be difficulty in introducing it again. After the first week or so the tube will be passed only at meal-times.

### Gastrotomy.

Gastrotomy is the operation of opening the stomach, usually in order to extract a foreign body which has been swallowed. The abdomen is opened, the stomach is drawn up into the wound and incised, the foreign body is extracted, the incision in the stomach is sewn up, and the abdominal wall sutured. There is nothing of particular importance to be said about the operation. The preparation and after-treatment are the same as for other abdominal operations.

### The Nurses' Clinic.

### HEAD INJURIES.

The nursing of "accident" cases is always most interesting, because of the variety of injuries, which, in consequence, demand a variety of treatment.

Take the case of a patient suffering from a head injury. Whether the injury appears serious or not at first, the patient must be carefully watched for symptoms which may afterwards develop, probably denoting the onset of something more serious than was anticipated in the first place.

It is always a good plan first to find out—if possible—how the accident occurred, for this often throws a good deal of light on the severity, or vice versa, of the injury. If, for instance, it is a fall from a height it is nearly sure to be more serious than an ordinary fall whilst walking, or from a slip; or, again, if the patient has fallen from a vehicle in motion he is more likely to be hurt badly than if he had fallen from one standing still. So first collect a few facts upon which to form some idea of the injury, and these, together with your own observation of the present and subsequent symptoms, will aid and suggest the treatment most suitable from a nurse's point of view, and thus adding also to your fund of knowledge and experience.

The first thing which suggests itself with regard to the treatment of these patients is the preparation of the bed. It is wise to protect the mattress with a long mackintosh sheet, or, if you are certain that it is nothing more serious than an ordinary scalp wound, it will only be necessary to place another small mackintosh above the usual one, in case of bleeding through the dressings.

Have ready hot bottles, for these patients are often very collapsed, and, if not, their feet should be kept warm. Also have ready a hot blanket, which you will put next to your patient after having removed all his own clothing, and put on a shirt opening down the back, being the garment most easy to put on and to remove without causing much movement. The patient will be best lying quite flat, unless he is subject to bronchitis or other chest trouble; in this case he will probably be best raised. If the accident has occurred to a labourer at work he will probably be dirty, but your zeal must not outrun your discretion; although he would certainly look better clean, it is just as well to let him rest and get over the shock a bit before you commence ablutions, which might prove more exhausting than beneficial. If possible, put your patient in a corner bed, or one which can be screened off for a time to ensure quietness, which is a great essential. As a rule no stimulants are given in these cases, but if the patient is severely collapsed the doctor may order a subcutaneous injection of strychnine or brandy.

In cases where the patient is suffering from contusion of the scalp an ice-bag or evaporating lotion is usually applied to the seat of injury, and often under this treatment the bruises or hæmatoma subsides. In the case of a scalp wound left to your care under the doctor's direction, the area round the wound must be shaved, then well wash the wound with soap and water, and finally syringe with some antiseptic lotion to insure the washing out of any particles of dirt, grit, and hair. If the wound is small it may be closed with adhesive strapping, or stitches may be put in, and the whole finally dressed with gauze and supported by a capeline bandage.

The foregoing treatment, for which the nurse is responsible, alludes chiefly to the less severe head injuries. In cases where the vault or base of the skull has been fractured the patient will be in a very serious condition, and if the fracture is depressed, and the patient's condition will permit, trephining will probably be done; but whether an operation is done or not, the treatment from the nurse's point of view will be practically the same at first. In the case of a fractured base there will be much bleeding from the ears, and probably the nose and mouth as well. The latter must be swabbed out to prevent the patient's throat getting full of the collection of blood and mucus, which he will not have the consciousness to eject himself, unless vomiting takes place, as it often does. The nostrils, too, must be cleansed and gently swabbed out with cotton-wool on forceps. The doctor may order the ears to be syringed out with weak carbolic or boracic lotion, after which they should be plugged with cotton-wool, which must be changed as often as it gets soaked with the hæmorrhage. In these cases the patient is generally unconscious, and there is therefore incontinence of both urine and fæces. It is a good plan to place pads—made of cotton-wool and compressed moss covered with

### THE NURSES' CLINIC—*Continued.*

gauze—under the patient's buttocks, so as to protect the bedding. Careful attention must be paid to the back, if it is to be kept in good condition. It should be washed with ordinary warm water and soap as often as it is needful to change the draw-sheet and pads; then rub in some spirit, and powder it well, or, if there is a distinct tendency to soreness, use some ointment. Unguentum zinci et benzoini is very good for this.

The diet for patients suffering from head injuries is usually restricted to fluids for the first day or two, then farinaceous and fish until the patient is again fit for the usual full diet; this, of course, refers to the less severe injuries only.

In the case of the more severe injuries and fractures—should the patient survive—it is often difficult to know how to feed him. If he is unconscious he will not be able to swallow, so that feeding by the mouth is out of the question. Where incontinence exists rectal feeding, too, will be of little use. The doctor may order nasal feeds, or give an œsophageal feed himself. It is important in all cases of head injuries that the bowels should act freely.

Calomel or some other smart purge is generally ordered to be given. The nurse must always report in these cases whether the aperient has been effectual.

However slight the injury may appear, it is wise to keep the patient in bed for a day or so. If he thinks it unnecessary, and is allowed to get up, you will no doubt find him ready to retire after sitting up a short time. Serious relapses are sometimes caused by the patient getting up too soon. Therefore it is best to be on the safe side and guard against the possibility of a relapse. The chief things to remember on revision are: (1) Be observant of any new sympton; (2) keep your patient very quiet and still; (3) keep him warm, especially the feet; (4) be careful of his back.

### The Nurses of the Park Hospital, Hither Green.

### INTERVIEW WITH THE MATRON. BY OUR COMMISSIONER.

Although the park at Hither Green is now only a memory, there are trees in the pleasant grounds of the hospital which attest the fact that it existed, and near the administrative quarters the Matron has a garden of her own. Nothing, indeed, struck me more on the occasion of my visit last week than the evidence outside the institution and within of the ample provision which is made for the comfort and convenience of the staff. The rooms which Miss Annie Thomas, the Matron, occupies are bright and tastefully arranged, and I found on subsequently inspecting the Nurses' Home, that in all respects it will bear comparison with similar buildings attached to leading general hospitals or Poor Law infirmaries. The conditions under which the nurses carry on their work will, however, be better gathered from the information readily supplied by the Matron in reply to my inquiries.

"First of all, perhaps you will tell me," I said, "the number of nurses employed in the hospital?"

"The normal staff consists of 40 charge nurses, 40 first assistant nurses, and 57 second assistant nurses. In addition to these, there are the matron, the assistant matron, the housekeeper, and the night superintendent, all fully-trained nurses. The number of beds is 548, and there are 16 scarlet fever wards, six diphtheria wards, two enteric wards, and twelve small isolation wards, making 36."

#### Training of Charge Nurses.

"Are the charge nurses all fully trained?"

"Yes; and practically, of course, they correspond to the sisters of the General Hospital. Charge nurses must, at least, be 25 years of age, and must hold a three years' certificate from a general hospital, or a Poor-Law infirmary which is a recognised school for nurses, and in which systematic instruction is given and tested by subsequent examination by an independent authority.

"I suppose, then, that you get your charge nurses from various institutions in the country?"

"Most of them come from the London Poor-Law infirmaries and well-known provincial hospitals. We also get a few from general hospitals in London."

#### Off Duty.

"How long are your charge nurses on night duty?"

"They change from night to day duty every two months. There is another point of difference. Our charge nurses are responsible for a pavilion consisting of two wards, one containing acute and the other convalescent cases. The junior charge nurses do relief work, so that the wards are never left without a trained nurse. With regard to off-duty time, it is the same here for each grade—two hours for meals each day, twelve hours each week, and one day a month. On her monthly day off duty a nurse may stay in bed for breakfast, and evening passes from 8.30 to 10 p.m. are granted. All nurses on night duty have three hours' leave a day for exercise, and two nights off duty a month."

"Is the annual leave for all nurses the same?"

"No. Charge nurses have four weeks' holiday and assistant nurses three weeks."

#### The Assistant Nurses.

"Your first assistant nurses, of course, are not trained?"

"They correspond to staff nurses in a general hospital. Nearly all have until lately been promoted from the second assistant nurses. If they come from outside they must have been engaged for one year in nursing at a general hospital or a Poor Law infirmary containing not less than a hundred beds, or have been nursing for two years at a fever hospital containing at least 100 beds. The latter is a new rule which only came into operation a month ago, and it may prove a considerable help, though I like to promote our own nurses when it is possible."

"The second assistant nurses," continued the Matron, "who must be at least 22 years of age, come as probationers, previous training not being necessary, and they must serve two years before they can become first assistant nurses to the satisfaction of the Medical Superintendent and the Matron."

#### Special Features of Work.

"And neither of these can become charge nurses without a three years' certificate of general training?"

"That is absolutely essential. Some of the assistant nurses who go away to be trained come back to us. We get the most useful charge nurses in that way, because the hospital-trained nurse who has not had fever experience has so much more to learn. For instance, she knows little about syringing the throat or ears, and less of nasal feeding. Another point is that she has very little knowledge of ward administration. The difference in the work is really very considerable."

"Do you think it is harder?"

"No, but more administration is required. Thus, in a general hospital the nurses have little to do with the clothing of the patients. Here we supply all the clothing of the patients the whole of the time they are with us. The clothing of patients, nurses, and domestic staff is all made on the spot, in the needle-room. Then, again, great care has to be taken in disinfecting and cleaning generally. In a general hospital a charwoman comes in for a few hours, but our ward maids are constantly engaged in scrubbing and cleaning. Indeed, fresh air and soap and water are a very important part of our treatment."

### The Diet.

"I suppose that another point with reference to which exceptional care must be taken is that of diet?"

"Certainly; we expect the nurses to be very regular with regard to their meals. It is of the utmost importance that they should keep themselves in good health, and if they fail to take their food they are expected to report themselves to the doctor."

"The matter of diet is so interesting, under the circumstances, that perhaps you could give me some details as to meals?"

"Here is a nurses' bill of fare for one week. You will see that at breakfast it varies every morning: cold boiled ham on Sunday, boiled eggs on Monday, fried bacon and eggs on Tuesday, smoked haddocks on Wednesday, cold boiled ham or bacon on Thursday, kippers on Friday, and fried eggs and bacon on Saturday. Porridge is served every morning. Dinner on Sunday consisted of cold roast beef, beetroot, celery, baked potatoes, apple tart, and custard and dessert. On Monday, hot roast mutton, potatoes, brussels sprouts, and milk pudding; Tuesday, hot roast beef, potatoes, and tomatoes, stewed pears, and baked cornflour pudding; Wednesday, hot boiled pork and stewed rabbit, and parsnips, baked apples, and rice and dessert; Thursday, hot boiled mutton, onion sauce, potatoes, turnips, and raisin puddings; Friday, fish and steak pies, potatoes, greens, and treacle-tarts; and Saturday, hot roast beef, potatoes, carrots, and tapioca pudding. At supper they always have a different kind of cold or hashed meat, with soup and bread and cheese; lunch, between 9 and 10 A.M., consists of coffee, bread and butter, bread and jam; and at tea cake is provided once a week, in addition to the bread and butter and jam. The dessert on Sundays and Wednesdays varies according to the fruits in season. The night nurses take on duty with them for their midnight meal a portion of raw meat, either chop or steak, with two uncooked vegetables."

"I imagine that you do not get any complaints about the diet?"

"No, but we think it very important that everything possible should be done to induce the staff to take their meals regularly. Of course, in regard to infection, they are warned not to expose themselves unnecessarily. The moment a nurse says she feels unwell she is relieved from duty, and sees the doctor. But very few contract either scarlet fever or any of the other diseases, and then they are usually the younger members of the staff."

"Are there more nurses on duty in some wards than others?"

"Yes, in each scarlet-fever pavilion, which contains one acute and one convalescent ward—46 beds in all—there is a charge nurse, two first assistant nurses, and two second assistant nurses on day duty, and one charge nurse, one first and one second assistant nurse on night duty. In the scarlet-fever cot wards there is a charge nurse, a second assistant, and a first assistant nurse always on day duty in each, and a charge nurse and second assistant nurse on night duty. The enteric and diphtheria wards are staffed in the same manner as the cot wards. There are only 10 beds in each. The experience in nursing is really very good."

### The Nurses' Home.

"And judging from a glance round the Nurses' Home, the accommodation for them leaves little to be desired."

"As you have seen, each nurse has a bedroom to herself, with a fireplace. There are separate sitting-rooms and dining-rooms for the charge nurses and the assistant nurses, with very comfortable furniture. There is also a third room, which is called the library. As you know, we cannot have books from an outside library. We buy a number of books at intervals, and the nurses subscribe 6d. a month. We have a fair collection."

"How many bedrooms are there in the Home?"

"A hundred and eighty-nine. Two of the blocks consist of bedrooms only, and the third includes sitting and dining rooms. In order that the night nurses may be quite shut off for quiet and rest the middle and the west blocks are alternately used for their accommodation. The assistant matron, Miss Smyth, who was trained at the London Hospital, and who has been here since this building was opened, has her quarters in the Home."

"When was the hospital opened?"

"In 1897—three years before I came as Matron. I was at the Grove Hospital, Tooting, as assistant matron, at the time it was opened, and I have found that experience very valuable. I was trained at the Birmingham General Hospital, and was afterwards charge nurse at Brook Hospital, subsequently acting for nearly a year as night superintendent. The managers of the Metropolitan Asylums Board behave very considerately to their staff, which, I think, is a great encouragement to them."

### The Question of Over-staffing.

"It is sometimes said that you are over-staffed?"

"That is not the case. During our slack time we do not fill up vacancies, and, it must be remembered, that in that time there is a very considerable amount of annual leave taken. For about four years we have transferred nurses to other hospitals under the Board, and all the staff sign a contract agreeing to the transfer in case of need. Autumn and winter are our busiest seasons, and for more than six months we often have between five and six hundred patients. As another illustration of the amount of work going on, I may say that at busy times, when we have extra beds in the wards, we cater for more than 900 people, and that we wash, roughly speaking, in the laundry about nineteen and twenty thousand articles weekly."

### Salaries and Pensions.

"I should like to know something about salaries?"

"The charge nurses begin at £36 per annum and rise by increments to £40; the assistant nurses at £24, rising to £28; and the second-class assistants at £20, rising to £24. I am sorry to say that a very large number of nurses contract out of the Superannuation Fund."

"At what age are they entitled to a pension?"

"Not until they are 65, which is our age limit. We have one nurse here who has nearly reached the limit, and she is quite vigorous and capable. After ten years of service, at whatever age, if disabled, a nurse is entitled to a pension. She can also claim a pension at 60 if she has had 40 years' service."

"Have you any religious difficulty?"

"None at all. I regret that we have no chapel, but St. Swithin's church is very near, and the nurses are granted a pass, as a rule, every Sunday morning or evening alternately. Special arrangements are made for Roman Catholic nurses, of whom we have about a dozen. There is a well-appointed little chapel attached to the mortuary, where the dead can be seen by their friends."

### Class of Nurses.

"Do you think there has been a change in the class of applicants for admission to the fever hospitals?"

"Fever nurses have improved as much during the last 40 years as the nurses in general hospitals. They are no longer of the Sairey Gamp type, but are hard-working, trustworthy women who try to do their duty conscientiously. This change in the London fever hospitals is mainly due to the work of the Metropolitan Asylums Board. Many who intend to be fully-trained nurses come here first to gain a little experience, without quite the hard work which is the rule in a general hospital. Again, many of our nurses have gone from here to various training schools in London and the country. Since the new hospital was built we get quite a mixed class of patients. In the same ward we may have the child of a professional man, a tradesmen, or a Polish Jew.

### Disinfection Rule.

"What are your rules about disinfection?"

"Nurses upon going out for a few hours have to change their dresses, petticoats, and stockings, and if they sleep out they are required to change entirely, having previously taken a bath and washed their hair; of course, no article of uniform is allowed to be worn outside the hospital. Print dresses and aprons are strongly discouraged, because the public, knowing that these are worn inside the hospital, might form the erroneous idea that the clothing had not been changed. As I pointed out in the Home, each nurse has a separate wardrobe outside her bedroom, in which she is expected to keep her private clothing."

### Affiliation Suggested.

"Have you any suggestion to make which might tend to facilitate fever training among hospital-trained nurses, and be also a benefit to fever hospitals?"

"I think it would be an exceedingly good thing if the general hospitals could be affiliated to hospitals like the Park, so that during the training of their nurses they could come to us, say, for a period of six months. The experience would give them a good idea of fever nursing, and would show them how fever hospitals have progressed during the last twenty-five years."

### Incident in the Life of a Cottage Hospital Matron.

### RINGING UP THE FIREMEN.

Whilst having my solitary dinner a note was brought to me—I find that notes and visitors have a strange preference for arriving during dinner time—asking me to admit a girl "very ill." I inquired of the woman if she knew what was the matter, but she could only tell me that the doctor said she was too ill to be nursed at home, and it might be a bad chill or it might be typhoid fever.

I gave instructions for her to be brought in a cab that afternoon. About 3.30 a very big young woman arrived, looking dreadfully ill, and with a peculiar look in her eyes which to my mind meant trouble in store. Her mother said she had been "light-headed" at night, and before long the girl was talking a good deal, and appeared to be rambling. The doctor came, gave a few orders, looked for "spots," and left me.

My night nurse got up at 8 p.m., and after some supper we had a good look at our new patient together, and decided we did not like her appearance. By 10 p.m. I said I should not go to my room, but would lie on the couch on the landing to be at hand if nurse required help, for there was every sign of coming trouble. I got snatches of sleep, but very short ones; for the delirium was noisy, and scraps of conversation concerning "two nuts," "the milkman," and "those bananas up there" kept me supplied with food for reflection.

Soon after 2 a.m. nurse came and told me she hoped that the girl was getting a little quieter, and "Shall we have some tea?" But I said we would wait a bit, and then have some together.

Alas for the quiet time! In half-an-hour or so there was a terrific noise, the girl sprang round in bed, seized the little hand-bell on her locker, and lurched out of bed while ringing vigorously. I was there in an instant, but our united efforts were useless to restrain that great, strong, thick-set girl. It was like fighting with a young horse, and she raved more like a mad woman than any other delirious patient, male or female, that I had ever seen, though we used to have some rough, strong customers to deal with in my training days. It was an awful moment when that supposed "typhoid" stood by her bed, holding each of us tightly by the hair of our heads, while she screamed and raved. The only person not an invalid in the place was the servant, so to her I screamed at the top of my voice. She was at the other end of the landing, and I knew slept soundly. When I got free for a moment I flew to the electric bell which rang into the room next hers, kept my finger upon it for nearly a minute, and did not let go till the girl appeared, looking terrified and half asleep, but even then the bell appeared to know that something was wrong; it refused to leave off ringing, and so added to the general confusion. I sent the servant for "a policeman or anyone she could find," but did not hear her start, so we concluded that the struggle she had witnessed was too much for her, and that she must have fainted with fright. I therefore left nurse to struggle in the far corner of the ward while I threw up a window and rang the hand-bell loudly out of it, asking a passer-by to try and find me assistance.

Then my turn came, and once more I was firmly gripped, whilst nurse was free for a moment. I despatched her to go as quickly as she could to undo the front door, so that when "help" did come our rescuer could get in. But I began almost to feel as if this state of affairs was going on for ever. While she was gone, our patient fought and struggled till she got me the length of a long landing, up five steps towards my bedroom. There she fell on her back in an awkward corner by the door, kicking, screaming, and tearing my clothes. I felt I was getting exhausted, and could not hold out much longer, and nurse looked nearly as bad. Whilst my apron was being clutched at furiously, I managed with one hand to undo the buttons and to step back before she made another grab at my frock. Then I resorted to my last possible resource for summoning help. I broke the glass of my fire-alarm, and pressed the bell. It may have been only a few minutes, but it seemed to be hours before there was any sign of a stir in the little fire-station, which was exactly opposite, but at last there was a sound of voices. Then I called out that there was no fire, but that I needed immediate assistance, and as quickly as running feet could bring them in they trooped, seven firemen, armed with helmets and hatchets, and two men in plain clothes, whom I afterwards found were a police-inspector and a constable!

Oh, the relief! Nurse and I could have done little more of such battling. We were almost done up. Asking two men to hold the patient down, I got a blanket to put over her while nurse prepared the bed again, and the men carried her back to the ward, and we popped her in. In a very few minutes the doctor arrived, and when the tension was over we were able to join in a good laugh over the unusual proceedings. The patient had a strong sleeping draught, and that and the exhaustion sent her into a deep sleep for hours. The men dispersed, our belated tea was made, and a policeman remained, in case there should be more trouble. But fortunately peace reigned for the rest of the night.

For two or three weeks after this the poor girl lay chattering unconsciously, her temperature keeping alarmingly high long after the regulation "enteric" period, but at last our efforts were rewarded; it came down, and strength was gradually regained.

I now often meet my patient out and about looking the picture of health, but it will be a long time before I shall forget the one occasion on which I had to use the "fire alarm."

### Probationers in Small Hospitals.

BY A MATRON.

This is—and I think has been for some years—a much vexed question amongst the matrons of small hospitals, and although I have no hope of finding a solution to the difficulty, I think perhaps by airing it the combined experiences of those who have had it to contend with, and the ready resources of many workers in the nursing world, some suggestions may be made that will prove helpful both to the much tried matron and also to the probationer. In a busy provincial town with a working population one usually finds a good modern hospital and an efficient staff of medical men. The hospital in an emergency will probably accommodate thirty-five to forty patients, but the average number of occupied beds at one time throughout the year we will estimate at twenty-five.

Now it is quite impossible for the matron, however capable she may be, to manage simply with domestic help, she must have at least two nurses, one for day duty and one for night, each of whom must be fully trained and capable of acting efficiently during her temporary absence. Now comes the question of probationers: a fully-trained nurse, unless she takes a very small sole charge, say, a hospital of six or eight beds, objects to what she may call the "drudgery" of probationer's work; besides, when there are "bad cases" an influx of accidents, or many operations, it is impossible to get through the everyday work without more hands. The actual amount of nursing done by the matron cannot be relied on, as she has her housekeeping duties, correspondence accounts, and the numerous interruptions occasioned by the visits of the medical staff and the friends of the patients.

I think a hospital such as I have described requires at least two probationers, so that when the day nurse is off duty the two probationers are left with the matron. Now the question is, should such a hospital undertake to train probationers, knowing that at the end of one or two years if the probationer aspires to be considered a fully-trained nurse she must enter a training school as a "raw probationer?"

That much useful knowledge may be gained, even in a hospital containing only twenty-five patients I admit, but in what form can it best be utilised for the advancement of the probationer herself, as the certificate she will receive is practically valueless, and I think it is only fair to let her know this?

When this drawback is explained many would-be nurses (unless they are too young or too old for a training school) withdraw and either wait for a better opportunity or take up other work. So the supply to the matron is limited and she often has to fall back on an undesirable applicant, or is constantly worried by the dissatisfaction expressed by the probationer that the training on account of the small number of patients is not what she expected.

Moreover, many training schools object to taking probationers who have had previous training. This again adds to the difficulty and even those who do not openly object do so privately, to the discomfort of the probationer. It is not always easy to hide one's knowledge under a bushel, especially to a young enthusiastic nurse. However much she may try in a big hospital to feel and act as a "raw probationer," something will occur which by word or deed will show that she is a year or two in advance of her position, and this in some instances will be looked upon as an unpardonable offence.

One idea which has occurred to me is for the matron of a small hospital to co-operate with the matron of a training school in the district, who will undertake to receive those probationers who have satisfactorily completed a year's training in the small hospital irrespective of the age clause.

As a rule there is no doubt that a probationer should be at least three and twenty when she enters a training school, but many girls are compelled to take up their life work before that age, and I think that if a girl—even of nineteen—has passed through a year creditably in a small hospital and can be thoroughly recommended by the matron, an exception might safely be made; also, there are others who from circumstances are not free to take up the work till they are thirty, and these I think would be none the less likely to succeed if they began their work in a small hospital.

I also think that perhaps the matron of the small hospital might be willing to take any probationer who, though satisfactory in all other respects, may not be quite sufficiently robust for a large hospital, though it might be arranged for her to return in a year's time should her health improve. Many girls who are not physically robust, or who have not acquired powers of endurance will make in the end admirable nurses if they are, as it were, "let down easily their first year." Temperaments vary so much, and if those who have the authority and the gift of studying individuals have the opportunity of disposing to the best advantage of the material which comes before them, much lasting good can be done.

It naturally saddens a girl to be obliged to give up work which she has set her heart upon because "she is not strong enough," when possibly after the first year's breaking in—which in a small hospital is easier because the life is more homelike—she may grow stronger and learn how to endure. Moreover, the thought that she may eventually return to her former hospital will act as a stimulus.

I do not think that the matrons of either hospital would lose anything by this plan; in fact, I think that it might attract the class of women they would desire to have. The probationer in the small hospital would know her ultimate destination and would do her best to acquire the desired recommendation, and the matron of the large hospital would have some assurance that her probationers, having been tried, would become creditable nurses.

I am not at all sure that this plan will sound feasible to those who are competent to judge, but it may lead someone to suggest a more applicable scheme. My own experience, and that of matrons with whom I am intimate, assures me that the difficulty is a real one. Of course, if all small hospitals, say, of less than fifty occupied beds, could afford to take only trained nurses and keep an extra number of wardmaids the need for probationers in any but those possessing training schools would cease to exist; but, in nearly all hospitals, expense has to be studied.

I have been somewhat of a rover in the nursing world and have worked in hospitals containing only a dozen or twenty patients, also where there have been hundreds, and for the comfort of those probationers in small hospitals I would say, "Make the most of your opportunities, for there is much to learn, even in the smallest sphere." Personally, I know I have learned sometimes in a small hospital valuable hints which I could not have learned elsewhere.

### Everybody's Opinion.

[Correspondence on all subjects is invited, but we cannot in any way be responsible for the opinions expressed by our correspondents. No communication can be entertained if the name and address of the correspondent are not given as a guarantee of good faith, but not necessarily for publication. All correspondents should write on one side of the paper only.]

### AN APPRECIATION OF MISS BASTER.

"A Former 'Sister Benyon'" writes: On Saturday, the 17th, there passed from our midst one who had spent a long life in the service of others. Miss Anne Baster, whose death you announced last week, was one of the first few Nightingale nurses, who were trained at St. Thomas's Hospital after the Crimean War experiences had turned thoughts of serious women to the necessity of opening up some better way of nursing the sick and wounded. The present Hospital of St. Thomas was not built then, and the old building had been pulled down and the site sold to raise money for the erection of an institution more up to date. During the waiting period, the sick were nursed and the staff housed, somewhat inadequately, in temporary erections in the old Surrey Gardens. At that period well-trained nurses were a desirable novelty, and after a very short training Miss Baster was made sister of the men's medical wards, and before long was moved to Bradford Hospital as its matron. From there she went to fill the same office at the Royal Berks Hospital, Reading, a post she retained for twenty-five years. I worked with her as probationer for six months, and as sister for nine years. She was respected and beloved by all her nurses, and succeeded in inspiring in most of them her own high ideal of duty, and of the honour and privilege it was to spend one's life in the amelioration of suffering. She carried on her work very quietly, and was an enemy to all interviews, testimonials, and all such advertisements, and greatly discouraged the showy element that has crept too much into the nursing world in the present day.

### THE REGISTRATION OF NURSES.

"Nurse H. P." writes from Cliftonville Hydro, Margate: With reference to your article on the State Registration of Nurses, the importance of this Bill being fairly drawn up before attempting to pass it through Parliament cannot be overrated by nurses who have suffered from the misconduct and inefficiency of some of our number. I quite agree with Mrs. Fawcett that something must be done to protect the public and the nursing profession from the pseudo-lady nurse—who has probably originated from a ward-maid in hospital—being foisted on the public as a private nurse. It is this class which produces the oft-repeated phrase from the public: "I will never have a hospital nurse in my house again as long as I live," together with extraordinary details of her misconduct and neglect of her patient for her own pleasure. Would it not be possible to have an examining board of doctors, to whom nurses could present themselves for a written and practical examination, to be held in all the larger towns annually, who, if passed with satisfaction to the examining board, would be qualified by a certificate, duly signed, which could be used for the protection of the public when employing a private nurse. Another advantage would be the impossibility of rustiness in work after having left hospital some time, if the certificate only qualified for a definite number of years. I have been private nursing myself for three years in Scotland, but, discovering that hospital work was advancing by leaps and bounds in the meantime, while I was learning nothing new in medical work, I threw up a private case to take a certificate in electrical treatment and massage, both of which subjects have developed since I was in hospital. To my mind it is more important for a private nurse to be up to date in her work than to go about flourishing a three years' certificate, which does not always represent the best qualified private nurse, but which the public apparently have absolute faith in at the present time. Private nursing depends so much upon individuality that even a nurse with years of hospital experience and good certificates may fail utterly to make a success of it.

### ARE NURSES EXPECTED TO LEARN TOO MUCH?

"A Sympathiser" writes: I feel I ought to opologise for trespassing so far on your kindness, but perhaps in time we shall have aired our griefs sufficiently, or else settled them satisfactorily. Some members of the nursing profession seem to have taken for their motto, "Meekly wait and murmur not," and therefore we who murmur are called "grumblers." Now, nothing is further from my mind than grumbling, but if things can be improved we might as well try to have them so, for the sake of our successors. The position of nurses twenty years ago is not a standard till the end of time. The methods of nursing are different, the necessary qualifications of a nurse are different; so why quote the nurse of twenty years ago? No doubt twenty years hence we shall be still more advanced, and to obtain the post of matron will be advertised something like this: "Wanted a fully qualified and experienced nurse, for the post of matron. One holding certificates for massage, domestic economy, sick cookery, and maternity preferred. A certificate for dispensing would be considered an advantage. Previous experience in similar post essential. Candidates must not be under thirty-five nor over forty years of age." Great as this exaggeration may seem, it will bear comparison with the nurse of to-day and twenty years ago. "Advanced education" seems to apply to nurses as well as other professions. A question arose in these columns as to whether nurses learned too much. That question is worthy of consideration. The things absolutely necessary for a nurse to know are taught during her training, and extra branches, such as massage, should be optional. A nurse may be a great success in practice, yet a failure in theory. The complaint of "Justice" in last week's issue, that she is unfit for nursing, being over the age-limit, is truly deplorable. For my part, I do not know the exact age-limit, and I know of women still nursing close upon sixty years of age. Surely "Justice" can obtain a post as assistant nurse; that is, if she possesses a three years' certificate, which is absolutely essential in the present day. There is no chance without it, no matter what experience the nurse may have had. Three years' certificate is the "hall-mark." I fear "Justice," though an experienced nurse, lacks this qualification; if so, I sympathise with her. Take for another example the fever nurse. Where is she in the present day? She may have nursed long and successfully all types of fever, yet she is not considered capable of holding a position above an assistant nurse. And why? Because she does not possess the "hall-mark" of a three years' general training. Is not this rather rough on the nurse of ten or twelve years' standing? Now that they realise the essential qualifications necessary for advancement it is too late for them to begin at the beginning. Those who thought themselves qualified ten years ago find that they are not considered qualified to-day, and the kindly, energetic, experienced nurse without the "hall-mark" is not given a moment's consideration. Even in fever hospitals, therefore, fever training is of no use of itself, and the sooner fever nurses swallow this bitter pill the better. Nevertheless they deserve some consideration for their experience, and should be qualified to nurse fever and hold the position of charge nurses, as they possess a fever certificate.

### NURSES AND THEIR GRIEVANCES.

"A CLERGYMAN'S DAUGHTER" writes: It is with great interest that I have read the discussions on nurses and their grievances. Do these few grumblers out of the large number of nurses think that they are the only "overworked" pros.? At present I am neither matron nor sister, but just finishing my three years' training, and it certainly has been a very happy three years, although I have had to work hard very often, and in a rush have been on duty from 7 A.M. till after 1 A.M. the next morning. As to night nurses, one correspondent said, "There is nothing to do between 9 P.M. and 12 A.M." May I ask where she trained, because I find that the four-hourly things fall due at 10 P.M.? Has she never had a bad case? No poultices to make, no fomentations to change, no medicines nor stimulants to give, no four-hourly charts? I often do not sit down till after 12 A.M. Of course night duty is unnatural, but why not make the best of it? "Gip" thinks that she is the only one who has any anxiety or worry. I am senior nurse on night duty, with no sister, my own wards to look after, and the responsibility of the whole place; also the night bell to answer and to attend to casualties alone, unless they are of such a nature as to require the house surgeon. Then as to the meal, do not eat indigestible things or drink your tea in a hurry. I often do not sit down to a meal all night, nor my fellow nurse. "Sympathiser" writes about the bedrooms of night nurses. I have as noisy a room as anybody could have when on duty, but go to bed so tired that I naturally sleep through everything. I write this during my last night on duty (the last night duty of my training), but I am heartily sorry it is over. I admire "H. M. G.'s" letter, and quite agree with her about the night nurse. All my patients have told me how sorry they are to lose me. Why do not these grumblers give up nursing, as it seems so distasteful to them? Probably they were spoilt as girls, and are now too bad tempered to live at home, and cannot marry, so take up nursing, and try to make others as discontented as themselves. What an example to set before a new "pro." Nurses ought to be bright, happy, and contented. Thank heaven I was educated at Clewer, and taught to be contented with my lot, whatever it may be.

### TRAINED NURSES AND UNTRAINED WOMEN.

"BETA" writes: May I say a few words to a "St. Thomas's Nurse" about "Trained Nurses and Untrained Women"? I think it a great pity that she should blame the doctors, as I, for one, have always found them very particular. It is not always convenient for a doctor to enter into details with a nurse about her training, etc., but woe to her if she does not do her part as a nurse. I have particularly noticed during my nursing career that "nursery maids" have made delightful nurses, and even a "general servant" may prove, with training, to be a "born nurse"; and it is the "born nurse" we want in this sad world, not "made nurses" with no love for the work, and not aiming at anything higher or nobler than getting money. I wish I had been taught more in the way of work before my training eight years ago. What does it matter about the uniform? It is not the uniform that makes the nurse. What we want is love for everybody.

"A NURSE" writes: I was very much disgusted when I had finished reading "A St. Thomas's Nurse's" views with regard to outdoor uniform. Surely this nurse cannot have much respect for her fellow nurses if she imagines that only fully-trained nurses should wear uniform! Then why should this nurse be so hard on fever nurses? I consider a fever nurse a very noble woman, running risks that very few general nurses would care to do. With regard to the behaviour, wherever there is a large staff there will be found black sheep, but why paint all alike? If anyone is found guilty of misbehaviour in the hospitals where I have been, that person is instantly dismissed. Again, there are many nurses in fever hospitals who are reduced in circumstances and are obliged to take a post where they can receive a salary, and therefore cannot afford to enter a general hospital. If nurses would devote themselves more entirely to the sick and to their efforts to restore them to health, they would think less of their petty jealousies. I hope that the nurses will give their own view with regard to this subject. Is it not also a fact that nurses who are trained are sometimes as capable of playing pranks as the untrained woman?

"TRAINED NURSE" writes: I am glad to see that "A St. Thomas's Nurse" advocates nurses not wearing outdoor uniform. I left it off myself a long time ago, and find that a plain tailor-made coat and skirt and neat hat answer just as well. It is very little trouble to change the skirt when you are going out, and there is the advantage of keeping it cleaner than when worn out of doors. I have known so many instances of untrained women being mistaken by the public for professional nurses, simply because they have worn some kind of uniform, that it is quite time the genuine nurse discarded it. In district work, I know, uniform is convenient, but in any other branch of nursing it is not necessary, and I hope all who can will discontinue wearing it.

"GOOD FORM THOUGH IN UNIFORM" writes: May a nurse with general training, employed in a fever hospital, comment on the letter of a St. Thomas's nurse on "Trained Nurses and Untrained Women"? In reply to the statement giving the "thoughtlessness of doctors" as a reason why unqualified women are employed to the detriment of real nurses, surely the medical man, to whom powers of observation, sound judgment, and good taste are as essential as they are to a nurse, usually satisfies himself as to the training and capacity of the nurse he employs, nor would he or any sane person suggest that the "girl with three months' massage experience or the woman once a nursery-maid and general servant who was for some time assistant in a convalescent home" should have the charge of a ward of sick patients or the care of a serious private case. As to the nursery-maids in uniforms of all colours (might I ask through what kaleidoscope the writer has viewed them? personally, I have never seen all the colours of the rainbow in Mary Ann's outdoor garb), how can they take the bread out of the mouth of one who has passed years of toil in a large general hospital? Again, none but an imbecile would requisition their services in cases requiring the training and technical ability of the modern nurse. Our three years' training in a large hospital ought to develop not only technical ability, but what is an even greater asset in life, intelligence, conscientiousness, powers of observation, judiciousness, and self-control. Possibly there are benighted persons in this dark age who might prefer the woman once a nursery-maid and general servant endowed with these qualities to the trained nurse without them. As to the animadversions on the employées of a "fever hospital not far off," it is to be deplored that a graduate of a highly respected training-school should have given way, in a public journal, to comments which, it must be admitted, more than "pushing a mail-cart," more than "going an errand," more even than "donning outdoor uniform," lay the would-be critic open to a charge of "bad form."

### THE CALCULATION OF THE STRENGTH OF SOLUTIONS.

"NURSE A." writes: I was glad to see the article on the calculation of the strength of solutions, but I am sorry to say that I do not yet understand it. Your contributor observes: "If you take a gallon of 1 to 20 lotion you must add one gallon of water to produce 1 to 40, three times as much water to make 1 to 60, four times as much water to make 1 to 80. Thus you have it:—

| | | | | |
|---|---|---|---|---|
| 1 gallon of | 1 to 20 lotion | +1 | gallon of water | =1 to 40 |
| ,, | ,, | +3 | ,, | =1 to 60 |
| ,, | ,, | +4 | ,, | =1 to 80 |

But why is it not:—

| | | | | |
|---|---|---|---|---|
| 1 gallon of | 1 to 20 lotion | +1 | gallon of water | =1 to 40 |
| ,, | ,, | +2 | ,, | =1 to 60 |
| ,, | ,, | +3 | ,, | =1 to 80 |

[The contributor, to whom we have referred the matter, regrets that she overlooked the inaccuracy which "Nurse A." points out.—ED. THE HOSPITAL.]

### MISSIONARY NURSES.

Sister Barbara, writing from the Sisterhood of St. Thomas ye Martyr, Oxford, says: Seeing that you are sometimes asked questions respecting missionary training for nurses, I should like to say that we have been engaged for the last ten years in training for foreign mission work, and can offer (as we require also) satisfactory references. Ladies trained here are now working in India, South Africa, and Japan. Nurses are specially welcomed, the certificate of the Central Midwives Board being particularly valuable for India and South Africa.

### LINSEED-MEAL POULTICES.

"A Matron" writes: I am anxious to get the advice of other matrons as to the making of linseed-meal poultices. I have always understood—and been taught—that the only accepted way was the following: Get all necessary things together, pull out tow to the required size, put boiling water into poultice bowl to warm it thoroughly, then pour that water off into some deep bowl or large cup with the spatula standing in it, pour necessary quantity of boiling water into poultice bowl, sprinkle meal in until the required consistency, spread quickly on prepared tow with the spatula, which should be occasionally dipped into the hot water so that it will run smoothly over face of poultice, a little olive oil to prevent its blistering, and gauze over all. Lately I have had two amongst my staff nurses, who have just completed their three years' training, whom I found putting in the meal first and then adding the water—they expressed great surprise at being told that I considered it wrong.

### Appointments.

[No charge is made for announcements under this head, and we are always glad to receive and publish appointments. The information, to insure accuracy, should be sent from the nurses themselves, and we cannot undertake to correct official announcements which may happen to be inaccurate. It is essential that in all cases the school of training should be given.]

Biggleswade Isolation Hospital.—Miss Margaret Purves has been appointed charge nurse. She was trained at Burnley Infirmary, Lancashire, and has since been charge nurse at the Parochial Hospital, Dundee, district nurse in Ross-shire, and staff nurse at the Baguley Sanatorium, Timperley, Cheshire.

Bishop Stortford Infirmary.—Miss J. B. Everett has been appointed superintendent nurse. She was trained at the Cancer Hospital, Fulham Road, London, and the Southwark Infirmary, East Dulwich. She has since been charge nurse and superintendent nurse at the Ipswich Infirmary. She holds the certificate of the Central Midwives Board.

Children's Hospital, Church Street, Kensington.—Miss H. E. Martin has been appointed staff nurse. She was trained at Dr. Steevens' Hospital, Dublin, and has since been on the private staff.

City Hospital North, Liverpool.—Miss C. Smith has been appointed night superintendent. She was trained at the Infirmary, Horton Lane, Bradford. She has since been ward sister at the City Hospital North, Liverpool, and she has also been attached to the Victorian Nursing Home, Harrogate. She holds the certificate of the Central Midwives Board.

David Lewis Epileptic Colony.—Miss Florence M. Smith has been appointed matron. She was trained at the Western Infirmary, Glasgow, and for district nursing at Scottish branch of Queen Victoria's Jubilee Institute at Edinburgh. She has since been nurse matron at Keswick Cottage Hospital, assistant matron at Roxburgh District Asylum, and matron of Ancoats Hospital Convalescent Home.

Deanhouse Workhouse, Holmfirth.—Miss Edith Blackburn has been appointed charge nurse. She was trained at Leeds Union Infirmary.

Dumfriesshire and Galloway Royal Infirmary.—Miss M. F. Gordon has been appointed lady superintendent. She was trained at the Royal Infirmary, Edinburgh.

General Infirmary, Macclesfield.—Miss Ida Freakley has been appointed sister. She was trained at the Wolverhampton and District Hospital for Women, Wolverhampton, the Seamen's Hospital, Greenwich, and the Hospital for Women, Soho Square, London.

General Infirmary, Stafford.—Miss Edith Parkes has been appointed sister. She was trained at the Blackburn and East Lancashire Infirmary, and has since been sister at King's Lynn Hospital.

Hartley Wintney Union Infirmary.—Miss Emily Barnes has been appointed assistant nurse. She was trained at the St. Peter's Memorial Home, Maybury Hill, Woking, Surrey, by the Meath Workhouse Nursing Association.

Hospital for Women, Soho Square, London.—Miss Evelyn Hirst has been appointed staff nurse. She was trained at the Royal Halifax Infirmary, and for fever training at the Sanatorium, Astley, near Manchester.

Oxford Workhouse Infirmary.—Miss Amelia White has been appointed assistant nurse. She was trained at the Home for Confirmed Invalids, Herbert Park, London, N., by the Meath Workhouse Nursing Association.

Rochdale Infirmary.—Miss Mary C. Reid and Miss Maud Brockbank have been appointed sisters, and Miss Betty Ford staff nurse. Miss Reid was trained at Rochdale Infirmary, where she has since been ward sister; she has also been Queen's nurse at Bolton. Miss Brockbank was trained at the Royal Infirmary, Liverpool, where she has since been staff nurse; she has since been doing private nursing. Miss Ford was trained at Lewisham Infirmary, and has since been staff nurse at Victoria Hospital, Belfast, and private nurse at Acland House, Oxford.

Royal Victoria Hospital, Dover.—Miss Ida F. Howe has been appointed sister. She was trained at the City of Dublin Hospital, and has since been charge nurse at Bromley Cottage Hospital. She has also done private nursing.

Shoreditch Infirmary.—Miss L. A. Houston has been appointed assistant matron. She was trained at the Children's Hospital, Newcastle-on-Tyne, and the Westminster Hospital, London. She has since been sister at West Ham Infirmary and matron at the Cottage Accident Hospital, Ebbw Vale.

### Presentations.

David Lewis Epileptic Colony.—At the David Lewis Epileptic Colony, Alderley Edge, on March 21, the matron, Miss Stapely, was presented with a handsome "illuminated address" and a "silver tea service." The presentation was made by the medical superintendent, on behalf of the staff and colonists.

### BOOKS RECEIVED.

J. and A. Churchill.

Chavasse's "Advice to a Mother," 16th edition, by T. D. Lister, M.D.

Y. J. Pentland.

"Handbook of Obstetric Nursing," 5th edition, by F. W. N. Hamilton, M.D., and J. H. Ferguson, M.D.

The Scientific Press, Ltd.

"Simple Introductory Lessons in Midwifery," by M. Loane.

In a notice of "A Lecture upon Massage and How to Use a Galvanic Battery in Medicine" last week our reviewer erroneously described the author as senior physician at the National Hospital for the Paralysed and Epileptic.

### Practical Hints.

We welcome notes on practical points from nurses.

### GERMAN METHODS.

At a time when we are hearing much of German methods in many departments, it may be interesting to note the plan adopted in some parts of the Fatherland in order to promote the proper feeding of infants.

When a father goes to register the birth of his child he is given at the office a printed leaflet bearing instructions as to the general care and feeding of babies, warnings against the indiscriminate use of prepared foods, and pointing out those symptoms which warrant the calling in of medical advice. A carefully prepared table is added giving the quantity and necessary dilution of milk for different ages.

It is at the time when doctors' and nurses' visits have ceased and the inexperienced mother feels "stranded" that the use of this leaflet proves its worth.

To the young father going for the first time to the registration office, the German doctor says: "Do not throw away—as you will most likely be prompted to do—the paper which you receive; your wife will find it very useful."

It is well known by all who visit among our lesser educated classes how ready they are to believe in any medical information brought under their notice in the form of print. This is an easy means of disseminating most needful knowledge.

### Novelties for Nurses.

(By Our Shopping Correspondent.)

### NEW FURNITURE AND UNIFORMS.

(Messrs. Thomas Wallis and Co., Limited, Holborn Circus, London, E.C.)

There are few hospitals in London, and indeed few institutions that have not at one time or another been supplied with furniture and fittings by Messrs. Thos. Wallis and Co., Limited, Holborn Circus, London. All kinds of ward furniture are to be seen here—beds with extra strong springs and india-rubber castors; cots with an ingenious arrangement whereby the side not only lets down but slides underneath and fastens itself securely, so as to be out of the way of the physician's shins when he bends over the small patient; lockers, ward tables, either with polished tops or covered with American cloth; dinner wagons, also with rubber castors; invalid chairs, bed tables—in short, every article of furniture that is needed in a hospital ward. Furniture for the rooms of the staff is also to be found in every variety. Quite recently Messrs. Wallis have supplied 250 sets of furniture for the staff of the London Hospital, all of oak. These sets included among other articles a very convenient hanging cupboard, made to fit into a corner; being 8 feet in height, there was plenty of room for a cupboard at the top, say, for bonnets and another below which would accommodate boots and shoes. A nice little chest of drawers and looking-glass, a neat washhand-stand with small cupboard, and a table with drawer were also part of the set. In the case of sisters they were given a table with pedestal of drawers one side, and enjoyed the luxury of a pretty easy-chair, upholstered in green, with springs and a well-cushioned back. Furniture for sitting-rooms is, of course, to be had, with many varieties of comfortable chairs, into which a wearied nurse may sink with a sigh of extreme contentment.

Every detail of nurses' uniform is also supplied by Messrs. Wallis. They do not keep a large stock of ready-made garments, preferring to make to order, as this is found most satisfactory as a rule. A very favourite style this season is a quite plain sleeveless cloak, in navy or black, from 17s. 9d. A silk hood, detachable, can be supplied for this cloak at about 2s. 6d. extra. A popular cloak, too, is a circular one with under fronts, and silk-lined hood, detachable, from 21s. 6d. This cloak can also be had in shower-proof material. Then there is a neat little surgical cape in black cloth, lined throughout with scarlet flannel, from 11s. 6d. A sleeveless ulster, with detachable cape, can be had in cloth in all colours from 31s. 6d., or in cravenette material for 21s. 6d. Uniform dresses are a strong point with Messrs. Wallis, and are, of course, made to order. The prices run from 31s. 6d. for alpaca and all-wool French cashmere to regatta washing cloth at 16s. 6d. All-wool serge or grey beige can be had from 25s. 6d. There are some very fascinating nurses' caps at most moderate prices, the Sister Dora cap being only 9½d., or 1s. with strings. Bonnets ranging from 6s. 6d. for fine straw, trimmed velvet, with ribbon strings, shoes, cuffs, collars, belts, dressing-gowns, and all descriptions of underclothing can be obtained at Messrs. Wallis's, as well as chatelaines, leather wallets, and other accessories.

### TRAVEL NOTES AND QUERIES.

By our Travel Correspondent.

A Month in Paris (Hope).—See my answer to "Garde Malade" on March 24th for reasons against cheap pensions. The lowest terms would be about 5 or 6 francs a day. Five francs a day would come to £1 8s. 4d. per week. The hotels I quoted would charge about 35s. per week for a long stay. This would be about the difference which omnibuses and trains would cost you to go to the cheaper houses. Let me hear further.

Holidays in August (Cecile).—Yes, it is rather long ahead, but you are wise to make plans in good time. If you can afford the long railway fare and carriage at the other end I can give you a good address in Cornwall. Journey altogether, both ways third class, would be £2 18s. Let me hear if this would suit you.

A Fortnight in Belgium (Mrs. McNab). Many thanks for all the information, which I have filed, all personal recommendations are so valuable. In Belgium, if all is new to you, I should recommend the following round: Three days in Antwerp will be plenty (it is frightfully modernized), Hotel Grand Miroir, 56 Vieux Marché au Blé. Leave by an early train for Brussels, giving yourself time to see Mechlin on the way. At Brussels, Hotel de la Cathedrale, 17 Place St. Gudule. Stay there four days—devote one to seeing Louvain, some twelve miles distant; the finest Hotel de Ville in Belgium is there, with two grand churches. I should not advise a visit to Waterloo; it is all smoothed out like a cricket field, no interest left. Leave Brussels early for Ghent, spending the day at Alost *en route*. Hotel de l'Etoile at Ghent. Stay there two days. Leave early for Ypres, visiting Oudenard and Courtrai *en route* if trains fit. If you must miss either let it be Oudenard. At Ypres go to Hotel Epée Royale. One day will be sufficient, as all of interest centres round the Grande Place. Be sure to see it in the twilight. Then up to Bruges Hotel Panier d'or. Stay there as long as you can, and return home via Ostend, where there is nothing of interest to detain you.

Rules in Regard to Correspondence for this Section.—All questioners must use a pseudonym for publication, but the communication must also bear the writer's own name and address as well, which will be regarded as confidential. All such communications to be addressed "Travel Correspondent, 28 Southampton Street, Strand." No charge will be made for inserting and answering questions in the inquiry column, and all will be answered in rotation as space permits. If an answer by letter is required, a stamped and addressed envelope must be enclosed, together with 2s. 6d., which fee will be devoted to the objects of "The Hospital" Convalescent Fund. Ten days must be allowed before an answer can be published.

### Notes and Queries.

**REGULATIONS.**

**The Editor is always willing to answer in this column, without any fee, all reasonable questions, as soon as possible.**

**But the following rules must be carefully observed.**

1. **Every communication must be accompanied by the name and address of the writer.**
2. **The question must always bear upon nursing, directly or indirectly.**

**If an answer is required by letter a fee of half-a-crown must be enclosed with the note containing the inquiry.**

*Address Wanted.*

(200) Will you please give me the address of the Church Missionary Society who want nurses in China?—*M. F.* and *L. O. S.*

Write to the Secretary, 14 Salisbury Square, E.C.

*Male Nurse.*

(201) How can I become "certified." I have had plenty of experience?—*N. H.*

The only hospital which gives male nurses a certificate is the National Hospital for the Paralysed and Epileptic, Queen Square, Bloomsbury. Write for particulars.

*St. John's Nurses.*

(202) Can you kindly tell me if the St. John's Nursing Association was in any way connected originally or influenced in its formation by the order of St. John of Jerusalem, which had branches in Europe and England? Also, where I could get interesting data concerning the former, for a paper I am preparing?—*District Nurse.*

There are two organisations whose nurses are known as St. John's Nurses. The headquarters of one is St. John's House, 7 and 8 Norfolk Street, Strand, of whom the Lady Superintendent is the Sister Superior of St. Peter's Community; and the Community of the Nursing Sisters of St. John the Divine, 19 and 21 Drayton Gardens, South Kensington, S.W., of whom Miss Beaver is the Sister Superior. If you want information respecting either write to the addresses given.

*Home.*

(203) Can you tell me of a home in which an old lady (bedridden, old fractured femur) could be received on payment of from 8s. to 10s. weekly?—*A. D.*

You might apply to the Alexandra Home for Chronic Invalids, St. Peter's Road, St. Leonards-on-Sea; or St. Peter's Home, Mortimer Road, Kilburn, N.W.; or consult Burdett's "Hospitals and Charities."

*Midwifery Training.*

(204) Will you be kind enough to tell me where I could get free training in midwifery? I am very anxious to become a midwife, but cannot afford to pay a premium.—*Inquirer.*

Write to the Secretary, Rural Midwives' Association, 47 Victoria Street, S.W.; also to the Midwives' Institute, 12 Buckingham Street, Strand, W.C.; or advertise.

(205) I am a trained nurse, but would like a little maternity experience, though I cannot afford to pay the usual fees. I should be glad to know if there is any free training to be had in London, or for a small fee.—*Trained Nurse.*

See answer to *Inquirer.*

*Denmark.*

(206) Is there any opening for an English trained nurse in Denmark, and where can I apply?—*Dane.*

We much doubt if there be any opening, but write to the British Consul at Copenhagen, and inquire.

*Roman Catholic.*

(207) Will you kindly tell me if there is a Guild or Society for Roman Catholic nurses in England, and the address?—*E. H.*

We do not know of one. Perhaps some of our readers might help you.

*Vienna.*

(208) I should be much obliged if you could tell me the name of the best maternity hospital in Vienna, and the surgeon of the same.—*Nurse Nora O'N.*

Write to the General Hospital, Vienna for any information you require.

**Handbooks for Nurses.**

| | Post Free. |
|---|---|
| "A Handbook for Nurses." (Dr. J. K. Watson) ... | 5s. 4d. |
| "Nurses' Pronouncing Dictionary of Medical Terms" | 2s. 0d. |
| "Art of Massage." (Creighton Hale.) ... ... ... | 6s. 0d. |
| "Surgical Bandaging and Dressings." (Johnson Smith.) ... ... ... ... ... ... | 2s. 0d. |
| "Hints on Tropical Fevers." (Sister Pollard.) ... | 1s. 8d. |

Of all booksellers or of The Scientific Press, Limited, 28 & 29 Southampton Street, Strand, London, W.C.

### For Reading to the Sick.

LOVE GIVEN AND RECEIVED.

My God! I love Thee;—not because
I hope for Heaven thereby,
Nor yet because who love Thee not
Are lost eternally.
Not from the hope of gaining aught,
Not seeking a reward;
But as Thyself has lovèd me—
O ever-loving Lord!

*From the Latin.*

Love is the greatest thing that God can give us, for Himself is Love; and it is the greatest thing we can give to God, for it will also give ourselves, and carry with it all that is ours. Let our love be firm, constant, and inseparable; not coming and returning like the tide, but descending like a never-failing river, ever running into the ocean of Divine excellency, passing on in the channels of duty and a constant obedience, and never ceasing to be what it is, till it comes to what it desires to be; still being a river till it be turned into a sea, and vastness, even the immensity of a blessed eternity.

*Bishop Jeremy Taylor.*

God so loveth us that He would make all things channels to us and messengers of His love. Do for His sake deeds of love, and He will give thee His Love. Still thyself, thy own cares, thy own thoughts for Him, and He will give thee Himself. Ask for Himself, and He will take thee into Himself. Truly a secret, hidden thing is the Love of God, known only to them who seek it, and to them also secret, for what man can have of it here is, how slight a fortaste of that endless ocean of His Love!—*E. B. P.*

There was only One on earth Whose Spirit was responsive to the perfect love of our heavenly Father, and we know that no thought of fear ever darkened His path. In some degree it is so still, even to ourselves, with our feeble imperfect love: in proportion as we trust and respond to His love, although we can never fathom its depth, so do we overcome every fearful thought and keep our minds in peace.

*Mrs. Campbell.*

"To know that I have loved thee"—this is the Christian's crowning triumph—the love of Christ—and when the follower of the Lord is cast down with many sorrows, or laid, it may be, on the couch of weakness or the bed of pain, what sweeter music can echo in his heart than these accents from the heavenly shore, "to know that I have loved thee"; and the soul makes humble answer, with the Apostle of old, "Lord, Thou knowest all things, Thou knowest that I love Thee."—*M. E. Townsend.*

O Love, Who formedst me to wear
The image of Thy Godhead here;
Who soughtest me with tender care
Through all my wanderings wild and drear;
O Love, I give myself to Thee,
Thine ever, only Thine to be.

*Catherine Winkworth.*

